# Necessity of Bumped Kinase Inhibitor Gastrointestinal Exposure in Treating *Cryptosporidium* Infection

**DOI:** 10.1093/infdis/jix247

**Published:** 2017-05-24

**Authors:** Samuel L. M. Arnold, Ryan Choi, Matthew A. Hulverson, Deborah A. Schaefer, Sumiti Vinayak, Rama S. R. Vidadala, Molly C. McCloskey, Grant R. Whitman, Wenlin Huang, Lynn K. Barrett, Kayode K. Ojo, Erkang Fan, Dustin J. Maly, Michael W. Riggs, Boris Striepen, Wesley C. Van Voorhis

**Affiliations:** 1 Department of Medicine, Division of Allergy and Infectious Disease, Center for Emerging and Reemerging Infectious Disease,; 2 Department of Chemistry,; 3 Department of Biochemistry, and; 4 Department of Biochemistry, Biomolecular Structure Center, University of Washington, Seattle;; 5 School of Animal and Comparative Biomedical Sciences, College of Agriculture and Life Sciences, University of Arizona, Tucson; and; 6 Center for Tropical and Emerging Global Diseases and; 7 Department of Cellular Biology, University of Georgia, Athens

**Keywords:** Cryptosporidium, physiologically based pharmacokinetic model, gastrointestinal, drug development

## Abstract

There is a substantial need for novel therapeutics to combat the widespread impact caused by *Crytosporidium* infection. However, there is a lack of knowledge as to which drug pharmacokinetic (PK) characteristics are key to generate an in vivo response, specifically whether systemic drug exposure is crucial for in vivo efficacy. To identify which PK properties are correlated with in vivo efficacy, we generated physiologically based PK models to simulate systemic and gastrointestinal drug concentrations for a series of bumped kinase inhibitors (BKIs) that have nearly identical in vitro potency against *Cryptosporidium* but display divergent PK properties. When BKI concentrations were used to predict in vivo efficacy with a neonatal model of *Cryptosporidium* infection, these concentrations in the large intestine were the sole predictors of the observed in vivo efficacy. The significance of large intestinal BKI exposure for predicting in vivo efficacy was further supported with an adult mouse model of *Cryptosporidium* infection. This study suggests that drug exposure in the large intestine is essential for generating a superior in vivo response, and that physiologically based PK models can assist in the prioritization of leading preclinical drug candidates for in vivo testing.

## Summary

Physiologically based pharmacokinetic models for bumped kinase inhibitors suggest that in vivo anticryptosporidial efficacy is associated with gastrointestinal concentrations of drug.

*Cryptosporidium*, a genus of protozoan parasites distributed worldwide, causes severe diarrhea disproportionately affecting those with poor nutrition and/or immunodeficiency. The Global Enteric Multicenter Study (GEMS) found *Cryptosporidium* to be the second leading cause of moderate to severe diarrheal disease and the leading pathogen associated with death in 6–18-month-old children at sites in both Africa and Asia [1–3]. Furthermore, children who survive cryptosporidiosis have associated growth and development stunting known to contribute to increased all-cause mortality rates and developmental issues [4]. The sole drug for cryptosporidiosis, nitazoxanide, has only a 30% response rate in malnourished children and no efficacy in human immunodeficiency virus–infected individuals [5]. Improved therapeutics are the greatest hope in the near term for addressing death and disability from *Cryptosporidium* infection. Species and strain antigenic differences and a lack of cross-protection impedes the development of a broadly protective vaccine [6].

There are unique challenges in drug development for cryptosporidiosis in that *Cryptosporidium* is a protozoan parasite and is localized to the gastrointestinal (GI) tract. As a protozoan parasite, *Cryptosporidium* is not susceptible to antibiotics which target processes specific to bacteria. In addition, *Cryptosporidium* is more closely related to humans than to bacteria or viruses, and this increases the risk for potential drug toxicity. We have reduced host toxicity by developing bumped kinase inhibitors (BKIs) that selectively target calcium-dependent protein kinase 1 (*Cp*CDPK1) compared with host kinases. A subset of the BKIs have shown inhibitor constant (*K*_i_) values <1 nmol/L against *Cp*CDPK1 and inhibit the growth of *Cryptosporidium* in vitro with EC_50_ values <5 μmol/L against the parasite itself while demonstrating little toxicity to human cells or in preclinical animal models [7].

Although we and others have made progress in the development of selective inhibitors of *Cryptosporidium* growth, the pharmacokinetic (PK) parameters that predict efficacy are unknown for compounds addressing its distinct localization. PK/pharmacodynamic (PD) indices use PK parameters such as the maximal and average unbound drug concentration (*C*_max_, *C*_avg_) and area under the drug concentration–time curve (AUC), along with in vitro–derived efficacy parameters, such as the 50% minimum inhibitory concentration or EC_50_, to predict the in vivo efficacy of drug candidates [8]. In the antimicrobial field, these indices have been well established and are used to guide the selection of dosing regimens for drug classes such as β-lactams [9, 10] and fluoroquinolones [11–13]. For anti-*Cryptosporidium* drugs, not only are we unsure which PK/PD index should be used for predicting in vivo efficacy, it is unclear which tissue drug concentration will provide the information required for predicting the desired in vivo response.

Once inside the GI tract, *Cryptosporidium* attaches to and invades enterocytes, where it becomes enclosed in a parasitophorous vacuole that underlies the apical host cell plasma membrane facing the fecal stream. Unique membranous and cytoskeletal structures separate the parasite from host cell cytoplasm, and the level and direction of molecular exchange between them remains poorly understood [14–17]. Given the unique localization of *Cryptosporidium*, systemic exposure may not be required for BKI efficacy, and the in vivo efficacy of BKIs may correlate better with their GI exposure. Targeting this profile would be expected to minimize toxicity associated with systemic drug distribution. A similar strategy has been used for treating nonsystemic GI infections with rifaximin, a nonabsorbable rifamycin antibiotic [18]. For BKIs, a correlation between fecal concentrations of BKI and in vivo efficacy was reported elsewhere [7]. However, further work is required to clarify the correlation between BKI exposure in different parts of the GI tract and the observed in vivo efficacy. It is difficult to measure the concentration of a drug in solution along the GI tract.

In the current study, we applied computational modeling using Gastroplus software (Simulations Plus) to develop a physiologically based PK model to simulate systemic and GI BKI concentrations for 2 mouse models of cryptosporidiosis. To investigate how differences in compound pharmacokinetics may affect in vivo efficacy, we selected 8 BKIs that demonstrate similar potency against *C. parvum* in vitro but display divergent in vivo pharmacokinetics. Dose-normalized and simulated concentrations of BKIs predict the in vivo efficacy in a neonatal and adult mouse model of *C. parvum* infection. The information on the importance of GI drug exposure provided herein will be valuable for the entire research community currently engaged in developing therapeutics for cryptosporidiosis and other enteric diseases.

## METHODS AND MATERIALS

Information regarding the *C. parvum* oocysts, generation of BKIs, *Cp*CDPK1 enzyme inhibition*, C. parvum* growth inhibition by BKIs, plasma protein binding, and neonatal mouse model of *C. parvum* infection has been described in detail elsewhere [7]. Information on BKI solubility and permeability, Gastroplus modeling, the interferon (IFN) γ knockout (KO) mouse model, and measurement of BKI levels in the GI tract of mice is described in the Supplementary Material. All animal experiments were approved by the Institutional Animal Care and Use Committees at the participating universities (University of Washington, University of Georgia, and University of Arizona).

To predict the inhibition of *C. parvum* growth in the neonatal model of *C. parvum* infection, the dose-normalized *C*_max_ was used to predict the inhibition of parasite growth along with a sigmoidal *E*_max_ model as described in Equation 1:

$$Inhibition\;of\;C.Parvum\;Growth(\%)=\frac{E_{max}\cdot f_{u}[I]}{f_{u}[I]+EC_{50}}$$ (1)

where *E*_max_ is the maximum observed inhibition in vitro, [*I*] is the BKI concentration (*C*_max_ or *C*_avg_), *f*_u_ is the fraction unbound to proteins, and the EC_50_ is the BKI concentration that inhibited 50% of *C. parvum* growth in vitro. For all BKIs, the maximum observed inhibition in vitro was 100%. To predict the efficacy of BKI with simulated GI concentrations, the *C*_max_ and *C*_avg_ were determined for each section of the GI tract and used as the value for [*I*]. The *f*_u_ value was set to 1 for predictions for BKI GI concentrations.

To predict the efficacy in the IFN-γ KO mouse model, the natural growth of *C. parvum* and the inhibition by BKI were both incorporated into the model. The natural growth rate of *C. parvum* was determined by natural log-transforming the relative luminescence units (RLUs) observed in the vehicle-treated mice after infection and fitting the data with a linear regression. The slope of the line was used as a rate constant to simulate the growth of *C. parvum* infection with a Malthusian growth model. For 5 days after administration of BKI, the RLU (oocysts shedding) was predicted with Equation 2:

$$\mathrm{Pr}edicted\;RLU=Ae^{kt}\cdot [1-(\frac{\frac{E_{max}\cdot f_{u}[I]}{f_{u}[I]+EC_{50}}}{100})]$$ (2)

where *A* is the amount of RLUs measured the previous day; *e* is the mathematical constant Euler's number; *k,* the growth rate constant; and *t,* the time in days. The predicted RLU values were log-transformed and fit with a linear regression. The slope of the predicted RLU values was compared with that observed in the BKI-treated mice by analysis of covariance, using GraphPad Prism software, and if the slopes differed significantly (*P* ≤ .05) the prediction was considered inaccurate.

## RESULTS

### BKI Pharmacokinetics and Predicted Efficacy in a Neonatal Mouse Model of *Cryptosporidium* Infection

Although the 8 BKIs used in this study share the same pyrazolo[2,3-*d*]pyrimidine scaffold, differing chemical side chains generate variances in plasma PK after an oral dose. When a single oral dose of each BKI was administered to mice, 30- to 60-fold differences between the *C*_max_ and AUC values were observed, respectively (Table 1). Given the lack of knowledge on the PK/PD relationship for BKI anti-*Cryptosporidium* efficacy, the single-dose BKI PK parameters and their respective EC_50_ values do not provide the information necessary for prioritizing compounds to test in preclinical animal models. Therefore, the BKIs were tested in a neonatal model of *C. parvum* infection to determine whether any systemic PK parameters are associated with in vivo efficacy.

**Table 1.** BKI Single-Dose Pharmacokinetics and Their Efficacy in the Neonatal Mouse Model

BKI	Dose, mg/kg	*C*_max_, μmol/L	AUC, μmol · min/L	Plasma Protein Binding, %	BKI EC_50_ for Inhibition of *C. parvum* Proliferation	Reduction in Neonatal Mouse *C. parvum* Infection, %
1294	10	0.8	430	84	2.7	56
1553	10	12.8	13 725	90	1.6	36
1318	10	6.5	2208	55	3.2	65
1369	10	0.3	128	37	2.3	70
1534	10	0.2	58	99	2.2	61
1649	10	4.9	6416	90	2.3	56
1556	25	7.0	2082	86	3.2	61
1557	10	0.9	67	84	5.5	33

Abbreviations: AUC, area under the curve; BKI, bumped kinase inhibitor; *C. parvum, Cryptosporidium parvum*; *C*_max_, maximal concentration; EC_50_, BKI concentration that inhibits 50% of *Cryptosporidium* growth in vitro.

Although each of the BKIs generated a significant reduction in *C. parvum* infection when tested in a neonatal model of cryptosporidiosis across the set, the in vivo efficacy had a large range of 33%-70%. Surprisingly, BKI 1553 displayed the highest *C*_max_ and largest AUC after a single oral dose, yet it had the least efficacy in clearing parasite infection, other than 1557 (Table 1). The BKI 1369 had the most efficacy in clearing parasite infection, although its *C*_max_ was the second lowest of the 8 BKIs, and its EC_50_ value for in vitro parasite growth inhibition was nearly identical to that of the other BKIs tested.

**Figure 1.**
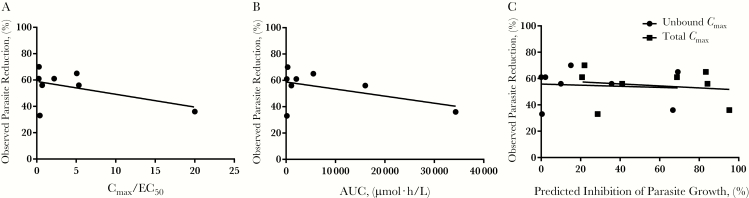
Predicting in vivo efficacy with dose-normalized bumped kinase inhibitor (BKI) maximal concentration (*C*_max_) and area under the curve (AUC). *A,* Observed reduction in a neonatal mouse model of *Cryptosporidium parvum* infection was plotted as a function of the dose-normalized *C*_max_/EC_50_ ratio for each BKI where EC_50_ is the BKI concentration that inhibits 50% of *Cryptosporidium* growth in vitro. There was not a significant association between the 2 values. *B,* Similar to the dose-normalized *C*_max_/EC_50_ ratio, the dose-normalized AUC was not a predictor of in vivo efficacy. *C,* Finally, sigmoidal *E*_max_ model was used to predict the in vivo efficacy, but neither the total nor the unbound dose-normalized *C*_max_ predicted the in vivo efficacy.

The single-dose *C*_max_ and AUC values were dose normalized and used to determine whether in vivo efficacy could have been predicted with BKI systemic PK values (Figure 1A), with the assumption that the BKI have linear PK. No significant association was observed between the efficacy and the dose-normalized BKI unbound *C*_max_/EC_50_ value, suggesting that the dose-normalized unbound *C*_max_ is not a predictor of in vivo efficacy. A comparable result was obtained when the total dose-normalized *C*_max_/EC_50_ was used to predict efficacy (data not shown). Similar to the *C*_max_ data, the dose-normalized AUC values did not predict the in vivo efficacy (Figure 1B). Finally, a sigmoidal *E*_max_ model that incorporated the dose-normalized *C*_max_ and the EC_50_ of each BKI was evaluated for its ability to predict the in vivo efficacy of each compound [19]. However, neither the unbound nor total dose-normalized *C*_max_ values predicted the in vivo efficacy (Figure 1C).

To overcome the inability to predict in vivo efficacy with systemic levels of BKI, the in vivo pharmacokinetics and physiochemical properties of each BKI were incorporated into a physiologically based PK model to simulate the BKI GI concentrations. BKI concentrations were simulated using the dosing protocol of the neonatal study, and the GI concentrations reported refer to the segments of the Gastroplus advanced compartmental absorption and transit simulation model. The mouse advanced compartmental absorption and transit model was adjusted as described in Supplemental Table 1 to represent more appropriately the reported physiology of a neonatal mouse [20]. For each GI segment, simulated concentrations of BKI in the lumen and the enterocytes were used to determine their respective *C*_max_ and *C*_avg_ values over the course of the neonatal efficacy study (Supplemental Table 2).

**Figure 2.**
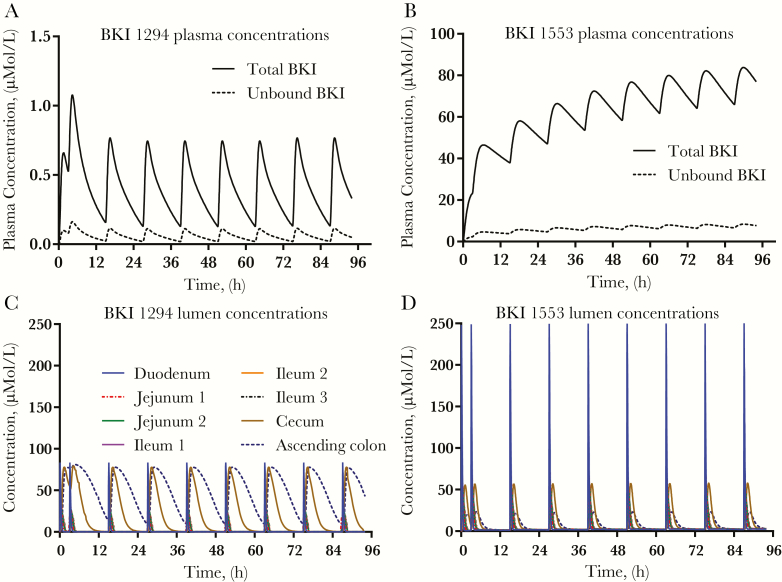
The full systemic and gastrointestinal (GI) bumped kinase inhibitor (BKI) concentration versus time curve was simulated for each BKI concentration over the duration of the neonatal mouse study. Gastroplus software was used to generate a physiologically based pharmacokinetic model to simulate drug dissolution, absorption, distribution, and elimination for each BKI; BKI 1294 (*A, C*) and 1553 (*B, D*) are shown as examples. *A, B,* Simulated total (*solid lines*) and unbound (*dashed lines*) BKI plasma concentrations. *C, D*, Exposure profiles in the lumen of the GI tract. Each line symbolizes a section of the Gastroplus advanced compartmental absorption and transit model. Because the plasma half-life of BKI 1553 is longer than that of BKI 1294, the systemic accumulation is greater for BKI 1553 over the duration of the study. In the GI lumen, whereas BKI 1294 exposure is predicted to be maintained between doses in the cecum and the ascending colon, BKI 1553 levels are predicted to rapidly rise and fall in each section of the GI tract after each dose, producing long periods with little BKI 1553 exposure between dosing.

Representative GI concentration versus time profiles for the BKIs are shown in Figure 2 for BKIs 1294 and 1553. The *C*_max_ for BKI 1553 is predicted to be greater than for BKI 1294 in plasma and a majority of the GI tract, whereas the *C*_max_ for BKI 1553 in the cecum and ascending colon are predicted to be less than for BKI 1294. In addition, although BKI 1553 is predicted to have brief increases in concentration after each dose, BKI 1294 is predicted to remain in the cecum and ascending colon for an extended period. Therefore, the predicted *C*_avg_ values for BKI 1294 in these GI sections are much higher than those for BKI 1553.

**Figure 3.**
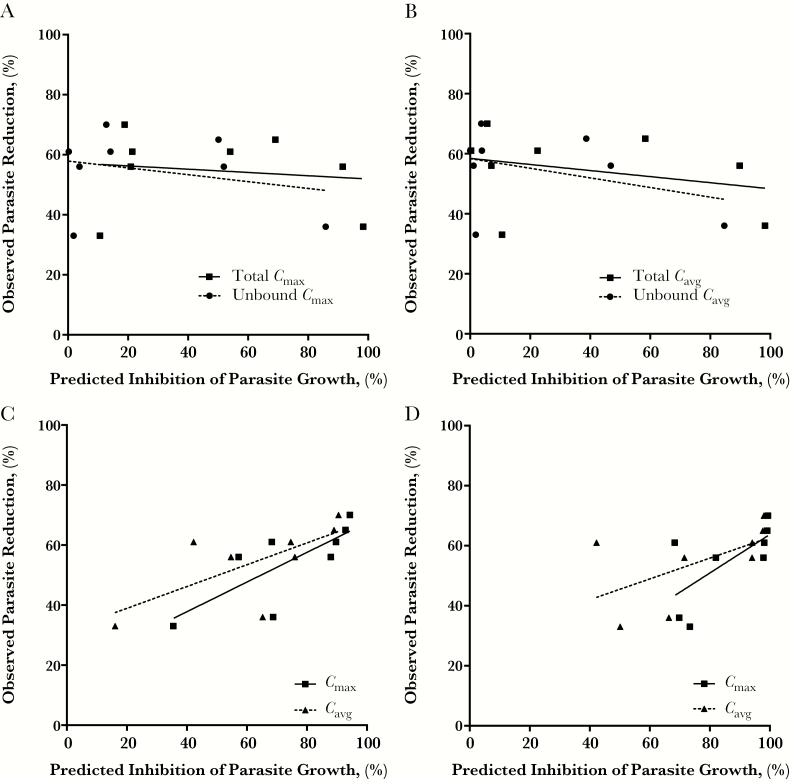
Simulated systemic and gastrointestinal (GI) concentrations of bumped kinase inhibitor (BKI) were used to predict the neonatal in vivo efficacy. *A, B,* As expected from the dose-normalized predictions, the model described with Equation 1 did not predict the in vivo efficacy when simulated plasma maximal concentration (*C*_max_) (*A*) or average concentration (*C*_avg_) (*B*) was used for the predictions. *C*, However, a significant (*P* < .05) association between predicted and observed efficacy was obtained when the ascending colon enterocyte *C*_avg_ (*R*^2^ = 0.60) or *C*_max_ (*R*^2^ = 0.47) levels were used with the sigmoidal *E*_max_ model. *D,* The ascending colon lumen concentration of BKI did not predict the in vivo efficacy.

The simulated concentrations for BKIs 1294 and 1553 in the plasma and the GI tract were used to predict efficacy in the neonatal mouse model. As expected, both the plasma *C*_max_ and the *C*_avg_ were not predictors of BKI in vivo efficacy, and accounting for plasma protein binding did not improve the prediction (Figure 3A and 3B).

The predicted BKI efficacy in each section of the GI tract was compared with the observed reduction in neonatal *C. parvum* infection (Supplemental Figure 2 and Figure 3). Similar to the efficacy predicted with systemic concentrations, BKI efficacy was not predicted using simulated concentrations in the duodenum, jejunum, or ileum (Supplemental Figure 2). However, when BKI concentrations in the enterocytes of the ascending colon were used to predict the in vivo efficacy, there was a significant (*P* < .05) positive association between the predicted and the observed efficacy (Figure 3C). Furthermore, there was a nonsignificant trend between the predicted and observed efficacy when ascending colon BKI concentrations in the lumen were used for the predictions (Figure 3D). In addition, when BKI concentrations in the lumen of the cecum were used to predict the in vivo efficacy, there was a significant (*P* < .05) positive association between the predicted and observed efficacy (Supplemental Figure 2D).

### Adult Mouse Model of *Cryptosporidium parvum* Infection

**Figure 4.**
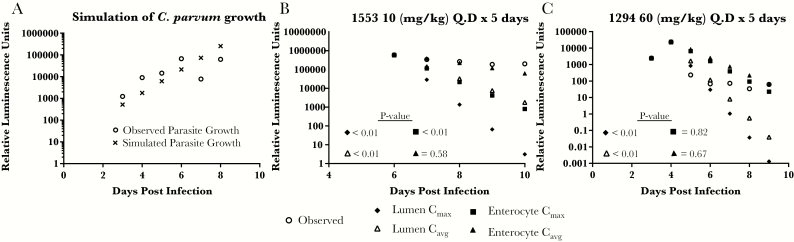
Simulating *Cryptosporidium parvum* growth in vivo and predicting in vivo efficacy with an adult mouse model of cryptosporidiosis. Adult interferon γ knockout mice were infected with nanoluciferase-expressing *C. parvum* oocytes. Therefore, oocyst excretion can be quantified by measuring luminescence after lysis of the fecal content. *A,* In vivo growth rate of *C. parvum* was simulated by log-transforming the observed relative luminescence in the feces of 3 vehicle-treated mice and fitting the data with a linear regression. *B, C,* The resulting growth rate was incorporated into the in vivo efficacy studies with bumped kinase inhibitor (BKI) 1553 (*B*) and 1294 (*C*). Ascending colon lumen or enterocyte levels were used to predict the inhibition of *C. parvum* growth with 5 daily oral doses of BKI 1553 (10 mg/kg) (*B*) or BKI 1294 (60 mg/kg) (*C*). The slope of the predicted relative luminescence unit (RLU) values was compared with that observed in the mice treated with BKI by analysis of covariance, and if the slopes differed significantly (*P* ≤ .05), the prediction was considered inaccurate. In vivo efficacy was accurately predicted with BKI 1553 enterocyte values for the average and maximal concentrations (*C*_avg_), but the lumen values overpredicted the in vivo efficacy (*B*). Similarly, the BKI 1294 efficacy was accurately predicted with the enterocyte *C*_max_ and *C*_avg_, but the lumen BKI 1294 levels over predicted the in vivo efficacy (*C*). For each study, 3 mice were used in both the BKI-treated and the vehicle-treated groups. Open circles represent results generated from the pooled feces of the 3 mice.

The ability to predict in vivo efficacy with BKI ascending colon concentrations was further evaluated in an adult model of *C. parvum* infection. Female IFN-γ KO mice were infected with transgenic *C. parvum* strain UGA1 expressing nanoluciferase (Nluc), and the infection was allowed to proceed for 3–5 days [21]. Infected mice were treated with oral doses of BKI 1294 (60 mg/kg), BKI 1553 (10 mg/kg), or vehicle control daily for 5 days, and oocyst shedding was measured by quantifying parasite-derived luminescence in the feces. To account for daily growth of parasites over the course of the study, the observed RLUs in the vehicle-treated mice were used to simulate the growth of the parasite over the course of the experiment (Figure 4A). On each day of the study, the simulated parasite level was based on both the growth of the parasite and the predicted inhibition by BKI [22–24].

The BKIs 1553 and 1294 *C*_max_ and *C*_avg_ in the lumen and the enterocytes of the ascending colon were used to predict the daily in vivo efficacy of the BKI over the duration of the study. These parameters were calculated for each day of the study using the simulated BKI concentration versus time profile over the 5 days of treatment (Supplemental Table 3). The observed and predicted luminescence was log-transformed, and linear regression was used to fit the data for the 5 days after treatment was initiated. The predicted efficacy was accepted as accurate if the predictions approximated what was observed and if there was not a significant difference (*P* > .05) between the slopes of the predicted and observed change in luminescence. When the simulated ascending colon luminal *C*_max_ and *C*_avg_ for BKI 1553 and 1294 were used to predict the in vivo efficacy, there was a significant difference (*P* < .05) between the predicted and observed RLU slopes over the course of BKI treatment, demonstrating that these values did not accurately predict the observed efficacy (Figure 4B and 4C). In contrast, the simulated enterocyte *C*_avg_ values for BKI 1553 and 1294 accurately predicted the rate of luminescence decrease, because the slopes of the predicted and observed luminescence did not differ significantly (*P* > .05) (Figure 4B and 4C). For BKI 1294, the simulated enterocyte *C*_max_ accurately predicted the in vivo efficacy as well (Figure 4C).

**Figure 5.**
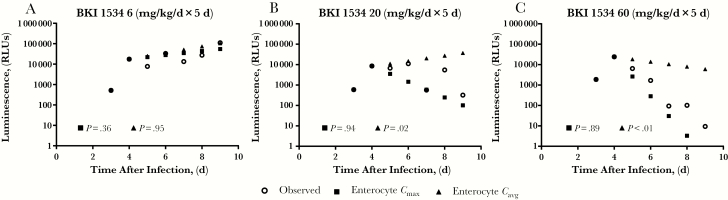
Simulated enterocyte bumped kinase inhibitor (BKI) concentrations in the ascending colon accurately predicted an in vivo dose response. Based on the accuracy of predicted efficacy with ascending colon enterocyte levels of BKI 1294 and 1553, the enterocyte maximal and average concentrations (*C*_max_ and *C*_avg_) for BKI 1534 were used to predict in vivo efficacy with 5 daily oral doses of BKI 1534 at 6 (*A*), 20 (*B*), or 60 (*C*) mg/kg. Predicted efficacy was compared with observed efficacy in an experiment where dosing was initiated on day 3 after infection (*open circles*). The slope of the predicted relative luminescence unit (RLU) values was compared with that observed in the BKI-treated mice by analysis of covariance, and if the slopes differed significantly (*P* ≤ .05) the prediction was considered inaccurate. Although the BKI 1534 enterocyte *C*_avg_ and *C*_max_ predicted the in vivo efficacy with daily dosing of 6 mg/kg (*A*), only the *C*_max_ predicted the in vivo efficacy at daily doses of 20 mg/kg (*B*) or 60 mg/kg (*C*). For each study, there were 3 mice in both the BKI-treated and the vehicle-treated groups. Open circles represent results generated from the pooled feces of the 3 mice.

The utility of the model was further evaluated with a dose escalation study of BKI 1534. Control vehicle-treated mice were again used to simulate parasite growth. Three days after infection, mice were treated once daily with a vehicle control or with BKI 1534 at doses of 6, 20, or 60 mg/kg (Figure 5). The ascending colon enterocyte *C*_max_ and *C*_avg_ values were calculated from the simulated concentration versus time profiles and used to predict the in vivo efficacy. For the 6-mg/kg study, both the enterocyte *C*_max_ and *C*_avg_ accurately predicted the lack of BKI efficacy. However, though the simulated enterocyte *C*_max_ accurately predicted the in vivo efficacy after doses of 20 or 60 mg/kg, the efficacy predicted with the enterocyte *C*_avg_ did not match the observed inhibition of parasite growth (Figure 5B and 5C).

### In vivo BKI Exposure Along the GI Tract

**Figure 6.**
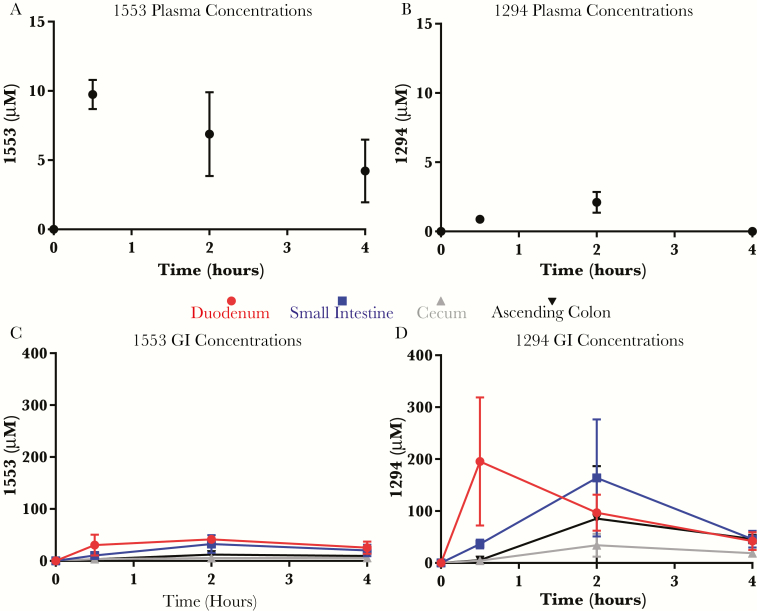
Plasma and gastrointestinal (GI) levels of bumped kinase inhibitor (BKI) observed after a single, oral dose. BKI plasma (*A, B*) and GI (*C, D*) concentrations were quantified 0.5, 2, and 4 hours after a single oral dose of BKI 1553 (10 mg/kg) or BKI 1294 (60 mg/kg). Despite a dose that was 6-fold greater, BKI 1294 plasma concentrations were much lower than BKI 1553 concentrations, in good agreement with previous results. However, systemic levels of BKI did not represent GI concentrations, and BKI 1294 levels were significantly greater in each of the 4 GI sections measured in this study.

To experimentally confirm that plasma concentrations of BKI are indeed not representative of their concentrations in the GI tract, BKI 1294 (60 mg/kg) and BKI 1553 (10 mg/kg) were each given as a single oral dose to 9 mice. The average plasma concentration of BKI 1553 was approximately 10 µmol/L by 0.5 hours, significantly (*P* < .05) higher than that for BKI 1294 at each time point (Figure 6A and 6B), consistent with findings of the initial single-dose PK study. Although the systemic levels of BKI 1553 were higher than those for BKI 1294, GI concentrations of the latter were higher over the course of the study (Figure 6C and 6D). Because BKI efficacy was associated with the ascending colon levels of BKI, the concentrations of BKI 1294 and 1553 in the ascending colon were compared using Student *t* test. At 4 hours, the average BKI 1294 concentration was significantly higher than that of BKI 1553 (46 vs 9 µmol/L, respectively; *P* < .01).

## DISCUSSION

The pathogen localization within its host’s organs and tissues is of utmost importance for the development of antimicrobial therapy. Simple models based on serum exposure alone are insufficient to predict cure, and knowledge of the specific site at which drug exposure is necessary is crucial to the development of novel therapeutics. As one of many examples, this is frequently discussed in the context of the blood-brain barrier and its impact on the treatment of different stages of African trypanosomiasis [25]. Information to build a reliable PK/PD index is not currently available for any class of anti-*Cryptosporidium* drugs; thus, we selected 8 BKIs with similar in vitro efficacy but divergent PK properties to identify PK characteristics associated with in vivo efficacy.

Plasma BKI levels do not predict in vivo efficacy, suggesting that systemic distribution of BKI is not required for anti-*Cryptosporidium* efficacy in mice. The lack of association between systemic exposure and in vivo efficacy is probably due to the GI localization of the parasite along the entire GI tract in both IFN-γ KO [26] and SCID/beige mice [27]. Dose-normalized PK values for the BKIs were used along with in vitro derived EC_50_ values to predict their respective efficacy in a neonatal model of *C. parvum* infection, but no associations were identified between any of the PK/PD indices and in vivo efficacy. Previous work in our laboratory identified BKIs lacking systemic exposure that generated an in vivo reduction in *C. parvum* infection [7], and a similar lack of association between systemic exposure and efficacy has been reported for the treatment of bacterial diarrhea by oral bicozamycin [28] and aztreonam [29]. Owing to the poor efficacy predictions generated with systemic levels of BKI and a correlation previously reported between high fecal levels of BKI and in vivo efficacy [7], we chose to investigate the importance of GI drug exposure for in vivo BKI efficacy.

Simulated large intestinal BKI levels accurately predicted the efficacy of the BKIs. Based on the parasite localization throughout the GI tract, it is likely that exposure of BKI in both the small and large intestines is crucial for efficacy, because BKIs with low exposure in the large intestine fail to clear the infection. It is likely that permeable compounds are quickly absorbed in the small intestine (eg, BKI 1553) and less permeable compounds are carried further along the GI tract (eg, BKI 1294), allowing them to maintain anti-*Cryptosporidium* activity. Although these initial BKI observations provided encouraging results, we tested the model with an IFN-γ KO adult mouse model of *C. parvum* infection to confirm the ability to predict in vivo efficacy with ascending colon BKI levels.

In agreement with findings in the neonatal mouse model, simulated ascending colon BKI concentrations accurately predicted the in vivo response to BKI treatment in the IFN-γ KO adult mouse model of *Cryptosporidium* infection. Although the neonatal mouse model provides only a single end point for assessing BKI efficacy, IFN-γ KO mice infected with Nluc-expressing *C. parvum* allow for daily measurement of *C. parvum* infection. This enabled development of a dynamic BKI efficacy model based on previous PK/PD modeling efforts for antibacterials and antitumor drugs [12, 22–24]. This model can account for differences in proliferation rates between *Cryptosporidium* strains or even in batches of oocysts.

We confirmed that simulated levels of BKI in the enterocytes of the ascending colon accurately predicted the in vivo efficacy of 3 BKIs. However, the importance of the *C*_max_ and/or the *C*_avg_ in the efficacy prediction was not consistent across the data set, so it is unclear whether the pharmacodynamics of the BKIs are time or concentration dependent. The GI BKI values measured in vivo were in agreement with the predicted Gastroplus values. The average maximum BKI 1294 and 1553 concentrations measured in the ascending colon were slightly higher than the values predicted for the enterocytes. This elevation could be due to residual BKI remaining after the lumen was flushed with phosphate-buffered saline and BKI in cells other than enterocytes. Although the measured ascending colon concentrations were higher than those simulated, the BKI 1553 values were significantly less than those measured with BKI 1294, correlating to the efficacy underperformance of BKI 1553 compared with BKI 1294.

Taken together, the results presented here provide strong support for optimizing drug exposure along the entire GI tract when treating *Cryptosporidium* infection. Therefore, adjusting the formulation to achieve the desired GI exposure profile will be essential for developing anti-*Cryptosporidium* therapies. Formulation strategies for such treatments may be especially challenging, because it is unclear how disease and altered GI transit times will affect drug dissolution and absorption. Mice do not develop clinical diarrhea with *C. parvum* infection, but studies in infected calves that exhibit diarrheal symptoms of cryptosporidiosis suggest that BKI exposure is not significantly altered by infection [30]. Malnourished children are an important focus of the current target product profile for *Cryptosporidium* drugs [31, 32], but it is unclear whether malnourishment will affect BKI pharmacokinetics, because previous studies have observed significant changes in antibiotic exposure in children with severe acute malnutrition [33–40].

In conclusion, to our knowledge this is the first study to investigate the importance of GI drug exposure for in vivo anti-*Cryptosporidium* efficacy. The results of this work have the potential to provide fundamental support for drug discovery programs in prioritizing lead drug candidates. In addition, these predictions can be tested in other GI infections and disorders in which large intestine epithelial cell delivery may be important.

## Supplementary Data

Supplementary materials are available at *The Journal of Infectious Diseases* online. Consisting of data provided by the authors to benefit the reader, the posted materials are not copyedited and are the sole responsibility of the authors, so questions or comments should be addressed to the corresponding author.

## Supplementary Material

Supplementary_Table1

Supplementary_Table2

Supplementary_Table3

Supplementary_Figure1

Supplementary_Figure2

Supplementary_Materials

## Notes

***Financial support.*** This work was supported by the National Institute of Allergy and Infectious Diseases, National Institutes of Health (grants R01AI089441, R01AI111341, and R01HD080670 to W. C. V. V. and grant R01AI112427 to B. S), the National Institute of Food and Agriculture, US Department of Agriculture (grant 2014–06183 to W. C. V. V.), the Bill & Melinda Gates Foundation (grants OPP1160955 to S. L. M. A. and OPP1134302 to B. S.), and the Program for Appropriate Technology in Health (grant DFI.1850-02-01291794-SUB to S. L. M. A.)

***Potential conflicts of interest.*** W. C. V. V. is the president of ParaTheraTech Inc, a company involved in developing BKIs for use in animal health. All other authors report no potential conflicts. All authors: No reported conflicts of interest. All authors have submitted the ICMJE Form for Disclosure of Potential Conflicts of Interest. Conflicts that the editors consider relevant to the content of the manuscript have been disclosed.
