# Supplementary material for: Necessity of Bumped Kinase Inhibitor Gastrointestinal Exposure in Treating Cryptosporidium Infection
Source: J Infect Dis. 2017 May 24;216(1):55–63. doi: 10.1093/infdis/jix247 (PMC5853285; doi:10.1093/infdis/jix247)
Supplement: Supplementary_Table1 [file jix247_suppl_supplementary_table1.docx]

**Supplemental Table 1: Neonatal Gut Physiology**

| Compartment | ASF | pH | Transit Time (h) | Volume (mL) | Length (cm) | Radius (cm) | SEF | Bile Salt (mM) |
| --- | --- | --- | --- | --- | --- | --- | --- | --- |
| Stomach | 0 | 2.98 | 0.08 | 0.370 | 0.2 | 0.3 | 1.00 | 0.0 |
| Duodenum | 64.36 | 4.87 | 0.15 | 0.013 | 1.1 | 0.1 | 6.63 | 2.8 |
| Jejunum 1 | 62.91 | 4.82 | 0.27 | 0.027 | 2.2 | 0.1 | 6.39 | 2.3 |
| Jejunum 2 | 64.69 | 4.82 | 0.23 | 0.023 | 2.2 | 0.09 | 6.06 | 2.0 |
| Ileum 1 | 67.6 | 4.81 | 0.13 | 0.013 | 1.5 | 0.08 | 5.79 | 1.4 |
| Ileum 2 | 71.57 | 4.81 | 0.11 | 0.011 | 1.5 | 0.08 | 5.58 | 1.2 |
| Ileum 3 | 76.43 | 4.81 | 0.09 | 0.008 | 1.5 | 0.07 | 5.36 | 0.1 |
| Cecum | 0.068 | 4.44 | 1.04 | 0.049 | 0.7 | 0.47 | 1.78 | 0.0 |
| Asc. Colon | 0.136 | 4.69 | 2.96 | 0.039 | 2.0 | 0.25 | 1.79 | 0.0 |

Absorptive Scale Factor (ASF), surface enhancement factor (SEF)

The ASF model was the Opt logD Models SA/V 6.1.
