# Supplementary material for: Necessity of Bumped Kinase Inhibitor Gastrointestinal Exposure in Treating Cryptosporidium Infection
Source: J Infect Dis. 2017 May 24;216(1):55–63. doi: 10.1093/infdis/jix247 (PMC5853285; doi:10.1093/infdis/jix247)
Supplement: Supplementary_Table2 [file jix247_suppl_supplementary_table2.docx]

**Supplemental Table 2: Simulated gastrointestinal (GI) bumped kinase inhibitor (BKI) pharmacokinetics in neonatal mouse model**

| BKI | Duodenum  Lumen: C_max_, C_avg_ (µM) | Jejunum 1  Lumen: C_max_, C_avg_ (µM) | Jejunum 2  Lumen: C_max_, C_avg_  (µM) | Ileum 1  Lumen: C_max_, C_avg_  (µM) | Ileum 2  Lumen: C_max_, C_avg_  (µM) | Ileum 3  Lumen: C_max_, C_avg_  (µM) | Cecum  Lumen:  C_max_, C_avg_  (µM) | Asc. Colon  Lumen: C_max_, C_avg_  (µM) |
| --- | --- | --- | --- | --- | --- | --- | --- | --- |
|  | Enterocyte: C_max_, C_avg_  (µM) | Enterocyte: C_max_, C_avg_  (µM) | Enterocyte:C_max_, C_avg_ (µM) | Enterocyte: C_max_, C_avg_ (µM) | Enterocyte: C_max_, C_avg_ (µM) | Enterocyte: C_max_, C_avg_ (µM) | Enterocyte: C_max_, C_avg_ (µM) | Enterocyte: C_max_, C_avg_ (µM) |
| **1294** | 90.2, 3.0 | 40.6, 2.9 | 32.3, 2.5 | 28.6, 2.4 | 26.4, 2.4 | 25.2, 2.3 | 80.1, 22.5 | 81.3, 48.7 |
|  | 15.6, 1.7 | 12.6, 1.4 | 11.1, 1.2 | 10.8, 1.2 | 9.8, 0.9 | 5.2, 0.9 | 9.1, 5.6 | 19.7, 8.5 |
| **1553** | 270.6, 11.2 | 80.6, 7.2 | 76.2, 7.5 | 45.7, 4.2 | 16.2, 3.4 | 15.5, 3.2 | 59.3, 5.3 | 23.6, 6.0 |
|  | 40.2, 2.5 | 13.3, 1.9 | 11.2, 1.8 | 9.5, 3.2 | 5.0, 3.1 | 4.8, 3.0 | 10.5, 2.8 | 4.7, 1.5 |
| **1318** | 394.7, 13.0 | 173.9, 12.7 | 139.4, 13.3 | 129.8, 13.3 | 122.5, 13.2 | 114.6, 12.7 | 287.5, 79.9 | 364.2, 135.5 |
|  | 48.9, 4.3 | 28.7, 4.1 | 25.5, 4.3 | 23.8, 4.3 | 22.3, 4.3 | 20.5, 4.2 | 10.4, 9.2 | 41.7, 25.9 |
| **1369** | 336.0, 10.1 | 146.3, 9.1 | 116.4, 9.2 | 106.6, 9.0 | 98.9, 8.8 | 91.1, 8.3 | 271.0, 69.8 | 349.4, 116.7 |
|  | 45.7, 2.1 | 24.8, 1.9 | 20.9, 1.9 | 18.9, 1.9 | 17.4, 1.8 | 15.7, 1.8 | 1 | 38.3, 22.1 |
| **1534** | 187.6, 28.5 | 149.6, 4.4 | 45.7, 1.9 | 14.5, 1.1 | 10.1, 0.9 | 9.1, 0.8 | 118.8, 29.2 | 114.6, 35.4 |
|  | 291.8, 6.3 | 25.2, 0.9 | 7.8, 0.3 | 2.7, 0.2 | 2.0, 0.2 | 1.7, 0.1 | 14.4, 5.9 | 20.0, 6.8 |
| **1649** | 4662.0  75.1 | 777.4, 21.0 | 155.3, 6.6 | 48.7, 3.6 | 19.3, 2.6 | 9.1, 2.6 | 13.6, 4.1 | 10.5, 5.8 |
|  | 629.6, 19.6 | 114.7, 5.9 | 24.2, 3.0 | 8.7, 2.4 | 4.3, 2.2 | 2.9, 2.1 | 2.6, 2.4 | 3.1, 2.8 |
| **1556** | 1116.0, 19.5 | 91.9, 3.0 | 28.0, 1.5 | 13.3, 1.0 | 7.7, 0.8 | 7.0, 0.7 | 36.1, 9.8 | 33.7, 11.8 |
|  | 179.4, 4.2 | 16.2, 0.7 | 5.7, 0.4 | 2.9, 0.3 | 1.8, 0.3 | 1.7, 0.2 | 6.4, 2.0 | 6.9, 2.3 |
| **1557** | 528.1, 10.5 | 46.8, 1.6 | 13.6, 0.8 | 9.0, 0.6 | 5.5, 0.4 | 4.0, 0.4 | 16.6, 4.8 | 15.2, 5.5 |
|  | 84.2, 2.3 | 8.2, 0.3 | 2.7, 0.2 | 1.8, 0.1 | 1.0, 0.1 | 0.8, 0.1 | 2.9, 0.9 | 3.0, 1.1 |
