# Supplementary material for: Necessity of Bumped Kinase Inhibitor Gastrointestinal Exposure in Treating Cryptosporidium Infection
Source: J Infect Dis. 2017 May 24;216(1):55–63. doi: 10.1093/infdis/jix247 (PMC5853285; doi:10.1093/infdis/jix247)
Supplement: Supplementary_Table3 [file jix247_suppl_supplementary_table3.docx]

**Supplemental Table 3: Simulated C_max_ and C_avg_ of bumped kinase inhibitors in the ascending colon lumen and the enterocytes of the adult mouse model**

| BKI dose | Asc. Colon  Lumen (µM)  C_max_ C_avg_ | Asc. Colon  Enterocyte (µM)  C_max_ C_avg_ |
| --- | --- | --- |
|  | 0-24 hours post dose 1 | 0-24 hours post dose 1 |
|  | 0-24 hours post dose 5 | 0-24 hours post dose 5 |
| 1553 10 mg/kg | 47.3 7.6 | 5.2 2.1 |
|  | 49.8 9.6 | 5.4 3.2 |
| 1294 60 mg/kg | 112.7 55.6 | 12.1 8.7 |
|  | 112.8 55.9 | 14.2 11.2 |
| 1534 6 mg/kg | 2.9 1.3 | 0.5 0.4 |
|  | 3.0 1.3 | 0.6 0.4 |
| 1534 20 mg/kg | 32.5 2.5 | 6.2 0.5 |
|  | 32.5 2.6 | 6.3 0.5 |
| 1534 60 mg/kg | 146.7 12.1 | 30.3 2.4 |
|  | 146.8 12.1 | 30.3 2.5 |
