# Supplementary material for: Necessity of Bumped Kinase Inhibitor Gastrointestinal Exposure in Treating Cryptosporidium Infection
Source: J Infect Dis. 2017 May 24;216(1):55–63. doi: 10.1093/infdis/jix247 (PMC5853285; doi:10.1093/infdis/jix247)
Supplement: Supplementary_Figure1 [file jix247_suppl_supplementary_figure1.pdf]

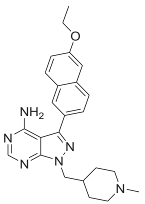

1294

C.parvum EC_{50}: 2.7μM
Solubility: >100, 82 μM
Permeability: 0.582 cm/s x10^4

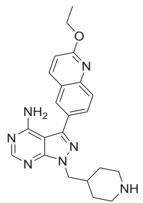

1318

C.parvum EC_{50}: 3.2 μM
Solubility: >100, 56 μM
Permeability: 0.407 cm/s x10^4

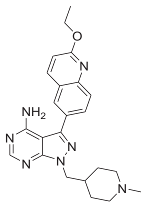

1369

C.parvum EC_{50}: 2.3 μM
Solubility: >100, 54 μM
Permeability: 0.558 cm/s x10^4

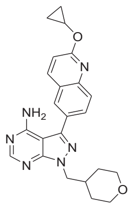

1557

C.parvum EC_{50}: 5.5 μM
Solubility: >100, 17 μM
Permeability: 1.222 cm/s x10^4

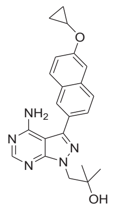

1553

C.parvum EC_{50}: 1.6 μM
Solubility: >95, 50 μM
Permeability: 0.837 cm/s x10^4

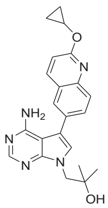

1649

C.parvum EC_{50}: 2.3 μM
Solubility: >100, 83 μM
Permeability: 0.758 cm/s x10^4

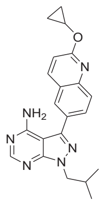

1556

C.parvum EC_{50}: 3.2 μM
Solubility: >53, 7μM
Permeability: 1.407 cm/s x10^4

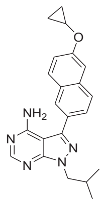

1534

C.parvum EC_{50}: 2.2 μM
Solubility: >95, 2 μM
Permeability: 1.548 cm/s x10^4
