# Supplementary figures and images for: Necessity of Bumped Kinase Inhibitor Gastrointestinal Exposure in Treating Cryptosporidium Infection
Source: J Infect Dis. 2017 May 24;216(1):55–63. doi: 10.1093/infdis/jix247 (PMC5853285; doi:10.1093/infdis/jix247)

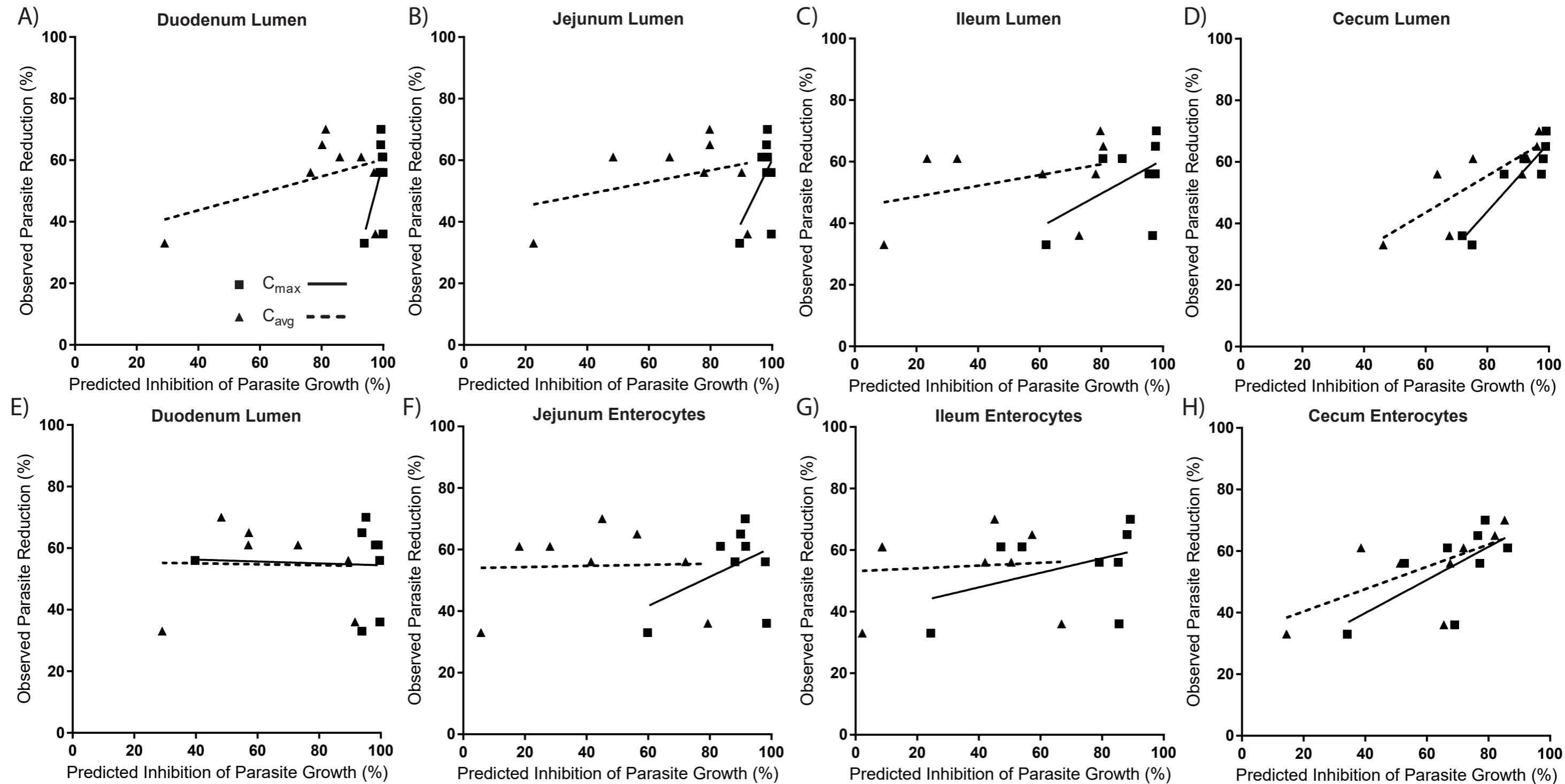

Supplement: Supplementary_Figure2 [file jix247_suppl_supplementary_figure2.pdf]
