# Supplementary material for: Necessity of Bumped Kinase Inhibitor Gastrointestinal Exposure in Treating Cryptosporidium Infection
Source: J Infect Dis. 2017 May 24;216(1):55–63. doi: 10.1093/infdis/jix247 (PMC5853285; doi:10.1093/infdis/jix247)
Supplement: Supplementary_Materials [file jix247_suppl_supplementary_materials.docx]

**Supplemental Methods and Materials**

***BKI solubility***

To characterize the aqueous solubility of each BKI, the solubility was measured at pH 2 and 6.5 as previously described [1]. Briefly, 1 μL of 20 mM dimethyl sulfoxide (DMSO) stock was added to 199 μL of buffer. Samples were vortexed and allowed to incubate at 25 °C overnight. The next day, each sample was centrifuged at 13800 rpm for 40 minutes at 25 °C. The supernatant was collected and 100 μL of the supernatant was added to 100 μL acetonitrile. Solutions were analyzed by HPLC/UV and were quantified based on a five-point calibration curve for each analyte.

***BKI permeability***

To characterize the permeability of the BKIs, five BKIs were tested with an in vitro Caco-2 culture system which has been shown to be a reliable predictor of intestinal permeability for lipophilic compounds such as these BKIs. The Caco-2 assay was performed as previously described with a few modifications [2]. Briefly, Caco-2 cells were obtained from American Type Culture Collection (Manassas, VA) and were cultured in DMEM supplemented with 10% FBS, 1% penicillin-streptomycin, and 1% MEM nonessential amino acids. After 10 passages, cells were seeded at 23,000 cells/well onto polyethylene-terephthalate, 0.4-μm pore-size filter inserts for 24-well plates (0.3 cm^2^ growth area) (Corning Inc., Corning, NY). After 21 days of culture, the cell monolayer integration was evaluated by measuring the transepithelial electrical resistance (TEER) using a Millicell electrical resistance system (ERS) (Millipore, Bedford, MA). A single filter insert without cells to determine background resistance. To test the permeability of control compounds, stock solutions of test compound (Lucifer yellow, 60 mM; metoprolol, 20 mM; and atenolol, 60 mM) were prepared in DMSO and diluted 200-fold in transport buffer to make compound solution (final concentrations were 300 μM, 100 μM, and 300 μM, respectively). The samples were analyzed with an EnVision Multilabel Plate Reader (Perkin Elmer, Waltham, MA) with excitation filter at 485 nm and emission filter at 538 nm. For BKIs, metoprolol, and atenolol permeability measurement, 0.32 ml of compound solutions was added to filter inserts, and 0.7 mL of receiver buffer was added to the basolateral compartment. Then 20 μL of the aliquots was withdrawn from the insert at 0 minutes, and 70 μL of the aliquots was withdrawn from the basolateral compartment and replaced with an equal volume of receiver buffer at 15–120 minutes. Then 20 μL of aliquots was withdrawn from the insert. Analytes were measured with an Acquity UPLC in tandem with a Waters Xevo TQ-S (Waters, Milford, MA). The apparent permeability coefficient (P_app_, cm/s) for each marker compound was calculated using equation 3:

$P_{app}= \frac{dQ}{dt}*\frac{1}{A*C_{0}}$ (3)

where dQ/dt is the rate of compound transfer (pmol/s) into the basolateral compartment, A is the surface area of the filter insert (cm^2^), and C_0_ is the initial concentration of the compound in the apical compartment. To mimic sink conditions, less than 20% of the compound was transferred across the cell monolayer for all experiments.

To determine if the ADMET predictor module (Simulations Plus Inc., Lancaster, CA) could reliably predict the permeability of the BKIs, the rat intestinal effective permeability (P_eff_) of the five BKIs were predicted in silico with and without the incorporation of the in vitro derived P_app_. There was a significant positive association (R^2^=0.95) between the predicted P_eff_ values with and without the incorporation of the in vitro P_app_. Based on the predictive power of the in silico predictions for the BKIs, the predicted P_eff_ without incorporation of P_app_ was used for each BKI and is reported in Supplemental Figure 1.

**Pharmacokinetic analysis of BKIs in mice and predicting BKI exposure with dose normalization**

Mouse oral PK studies were performed as previously described [3]. Briefly, three female BALB/c mice (10 -12 weeks old) were used in each group. With the exception of 1556, each group of mice received an individual BKI at a dose of 10 mg/kg body weight by oral gavage in 200 μL 70% Tween 80/30% ethanol diluted in saline. The only difference for mice administered 1556 was that the group received a dose of 25 mg/kg. Blood samples were taken by tail bleeding into heparinized tubes at designated time points and centrifuged to obtain plasma. The samples were frozen at -20°C. The plasma samples were prepared by adding acetonitrile + 0.1% formic acid with an internal standard. Calibration curves were generated by spiking standards of each BKI into mouse serum and preparing the standards along with test samples. The PK calculations of C_max_ and AUC were performed using Phoenix WinNonlin software (Certara, Princeton, NJ). To predict the C_max_ and AUC values for each BKI after a 25 mg/kg dose using dose normalization, the C_max_ and AUC of BKIs were multiplied by 2.5 except for 1556.

**Gastroplus Simulations**

In vivo BKI concentrations were simulated with the software GastroPlus™ V.9.0 (Simulations Plus Inc., Lancaster, CA), which uses a physiologically based advanced compartmental absorption and transit (ACAT) model with nine compartments corresponding to different segments of the digestive tract [4]. Differential equations are used to model the release, dissolution, and absorption of each BKI for each of the nine compartments. Compound specific parameters such as aqueous solubility and plasma protein binding were characterized and incorporated into the model [3]. Based on the accuracy of the simulated P_eff_ values without P_app_ input, the P_eff_ values generated by the ADMET predictor were used for the ACAT simulation for all eight BKIs. Predicted bile salt effects were included in the simulation for each BKI. In addition, the Gastroplus mean default value of 900 seconds for mean precipitation time was used for each compound.

To simulate the BKI concentrations for the neonatal efficacy study, the gut physiological parameters of Gastroplus’ “Mouse-physiological-fed” model were modified based on previously reported neonatal GI physiology (Supplemental Table 1) [5]. For the adult most efficacy study, the default Gastroplus “Mouse-physiological-fed” model was used. In addition, the weights of the neonatal and adult mice were set at 5 and 20 grams, respectively. Using the PKplus module within Gastroplus, a one or two compartment model was fit to the concentration versus time profiles that were observed after a single oral dose of each BKI (Supplemental Table 4). Based on the localization of *C. parvum* to the duodenum, jejunum, ileum, cecum, and ascending colon, the simulated luminal and enterocyte concentrations of BKI in each of these sections were plotted as a function of time. The resulting concentration versus time plots were used to calculate the BKI C_max_ and the C_avg_ concentrations over the course of the efficacy studies. For the neonatal study, the C_avg_ at steady state (C_ss_) was estimated using the equation C_ss_ = AUC_ss_/dosing interval (tau) with the assumption that AUC_0-inf_ = AUC_0-tau_. Gastroplus was used to generate a concentration versus time profile for both the plasma and GI BKI concentrations after a single oral 25 mg/kg dose in the neonatal mouse model. The AUC_0-inf_ was calculated by the linear trapezoidal method for the plasma and GI compartments using Phoenix WinNonlin. To estimate the C_avg_ for the neonatal study, the AUC_0-inf_ was divided by the dosing interval for the maintenance dose (every 12 hours) that was used in the neonatal efficacy study. Time to steady state will not only vary between the BKIs, but also between each section of the GI tract for the individual BKIs. However, for most BKIs, steady state is predicted to be reached within the first 24 hours of the study. For a few BKIs, steady state in the plasma or GI compartments will not be achieved by the end of the 94-hour study, and the estimated C_avg_ will overestimate the average drug concentration for the study. However, drug levels are expected to be very close to steady state levels by the end of the study. To predict the efficacy with the C_max_, the highest concentration observed over the 94 hour simulation was used to predict the efficacy.

For the adult mouse model, the AUC for each dosing interval of the study 0-24 hours, 24-48 hours, 48-72 hours, 72-96 hours, 96-120 hours was used to calculate the average concentration over each time interval (C_avg_=AUC/24 hours). The C_avg_ for each 24 hour period was used to predict the change in RLU for each 24 hour period. In addition, the C_max_ for each 24 hour period was also used to predict the BKI efficacy. These calculations were performed for both the lumen and enterocytes of the ascending colon in the adult mouse model.

**IFN-γ KO knock-out mouse efficacy model**

The efficacy of BKIs in an adult mouse model of *C. parvum* infection was tested as previously described [3, 6]. Briefly, all female IFN-γ KO mice (B6.129S7-Ifngtm1Ts/J, Jackson Laboratories), were infected by oral gavage (PO) with 10,000 Nluc expressing *C. parvum* oocysts in 100 μL DPBS for studies with 1534. For all other studies, mice were infected PO with 1,000 Nlucexpressing *C. parvum* oocysts. Vehicle control groups and BKI treated groups each contained at least three mice. For the first efficacy study, mice were dosed PO with 1294 (60 mg/kg) suspended in 200 μL vehicle or vehicle only once daily for 5 days beginning on day 4 post infection. For the second efficacy study, mice were dosed PO with 1553 (10 mg/kg) or vehicle only once daily for 5 days starting on day 6 post infection. Finally, for the dose escalation study, mice were dosed PO with 6, 20, or 60 mg/kg 1534 suspended in 100 μL vehicle or vehicle only once daily for 5 days beginning on day 4 post infection. On each day of dosing, stool was collected from each group and mice were moved to new cages. The stool samples were checked for luminescence on the day of collection and RLU readings were standardized to fecal sample weights. Background levels were obtained by collection and lysis of feces from uninfected mice dosed with vehicle only.

***Measuring BKI levels in the GI tract of mice***

Concentrations of 1553 and 1294 in the duodenum, small intestine, cecum, and ascending colon were measured after a single 10 mg/kg (1553) or 60 mg/kg (1294) oral dose. Nine mice were dosed PO with either BKI or vehicle. At 0.5, 2, and 4 hours, three mice in each group were sacrificed by cervical dislocation, and the duodenum, small intestine (jejunum and ileum), cecum, and ascending colon were immediately collected. Each section of the GI tract was flushed with Dulbecco's phosphate-buffered saline, weighed, and placed in storage at -80°C. On the day of homogenization, 0.9% NaCl was added to each sample to achieve 200 mg/mL tissue concentration and samples were homogenized using a handheld motorized homogenizer (Fisher Scientific, Waltham, MA) on ice. Tissue from vehicle control treated mice was homogenized and placed in a 96 well plate to be used as blanks or standards. As an internal standard, BKI 1318 was added to each sample to a final concentration of 2 μM. Calibration curves for 1553 and 1294 were prepared separately by adding 2 μL BKI to generate nominal concentrations of 0.01, 0.05, 0.1, 0.5, 1, 10, 30, 50, 100, and 200 μM. Calibration curves were generated separately for each section of the GI tract. Separately, 2 μL ethanol was added to each sample and blank. On the day of tissue extraction, 25 μL of each sample was added to a 96 well plate that contained 400 μL acetonitrile. The samples were mixed and centrifuged at 4,200 x g for 20 minutes. 25 μL of the supernatant was placed in a 96 well plate that contained 475 μL of 80/20 acetonitrile:water. The samples were mixed and centrifuged at 4,200 x g for 20 minutes. 100 μL of supernatant from each sample was placed in a 96 well plate, sealed, and analyzed by LC-MS/MS as previously described with a few modifications [3]. The settings for the Waters Xevo TQs were not changed, but the LC method was adjusted by increasing the flow rate to 0.5 mL/min with the following gradient: 90%-80% A over the first minute, 80%-10% A from 1 to 3.5 minutes, a further decrease to 5% A until 5 minutes, followed by an increase back to 90% A until 7 minutes. The solvents were A: water with 0.1% formic acid and B: acetonitrile with 0.1% formic acid. Sample concentrations were determined using the internal standard normalized calibration curves. To report tissue concentrations of BKI, one gram of tissue was estimated to be one mL of volume. Tissue BKI concentrations were compared using a Student’s t-test.

**References:**

1. Vidadala RS, Rivas KL, Ojo KK, et al. Development of an Orally Available and Central Nervous System (CNS) Penetrant Toxoplasma gondii Calcium-Dependent Protein Kinase 1 (TgCDPK1) Inhibitor with Minimal Human Ether-a-go-go-Related Gene (hERG) Activity for the Treatment of Toxoplasmosis. J Med Chem **2016**; 59:6531-46.

2. Yamaura Y, Chapron BD, Wang Z, Himmelfarb J, Thummel KE. Functional Comparison of Human Colonic Carcinoma Cell Lines and Primary Small Intestinal Epithelial Cells for Investigations of Intestinal Drug Permeability and First-Pass Metabolism. Drug Metab Dispos **2016**; 44:329-35.

3. Hulverson MA, Vinayak S, Choi R, et al. Bumped-Kinase Inhibitors for Therapy of Cryptosporidiosis. J Infect Dis **2017**.

4. Agoram B, Woltosz WS, Bolger MB. Predicting the impact of physiological and biochemical processes on oral drug bioavailability. Adv Drug Deliv Rev **2001**; 50 Suppl 1:S41-67.

5. Dehmer JJ, Garrison AP, Speck KE, et al. Expansion of intestinal epithelial stem cells during murine development. PLoS One **2011**; 6:e27070.

6. Vinayak S, Pawlowic MC, Sateriale A, et al. Genetic modification of the diarrhoeal pathogen Cryptosporidium parvum. Nature **2015**; 523:477-80.
